# Comprehensive small RNA-sequencing of primary myeloma cells identifies miR-105-5p as a predictor of patient survival

**DOI:** 10.1038/s41416-022-02065-1

**Published:** 2022-11-29

**Authors:** Kristin Roseth Aass, Tonje Marie Vikene Nedal, Siri Anshushaug Bouma, Synne Stokke Tryggestad, Einar Haukås, Tobias Schmidt Slørdahl, Anders Waage, Therese Standal, Robin Mjelle

**Affiliations:** 1 Centre of Molecular Inflammation Research, Department of Clinical and Molecular Medicine, Norwegian University of Science and Technology, 7491 Trondheim, Norway; 2 Department of Clinical and Molecular Medicine, Norwegian University of Science and Technology, 7491 Trondheim, Norway; 3 Department of Hematology, Stavanger University Hospital, 4011 Stavanger, Norway; 4 Department of Hematology, St. Olavs University Hospital, 7030 Trondheim, Norway; 5 Bioinformatics Core Facility—BioCore, Norwegian University of Science and Technology NTNU, 7491 Trondheim, Norway; 6 Department of Pathology, St. Olavs University Hospital, 7030 Trondheim, Norway

**Keywords:** Prognostic markers, Non-coding RNAs

## Abstract

**Background:**

Small RNAs (sRNAs), a heterogenous group of non-coding RNAs, are emerging as promising molecules for cancer patient risk stratification and as players in tumour pathogenesis. Here, we have studied microRNAs (miRNAs) and other sRNAs in relation to survival and disease severity in multiple myeloma.

**Methods:**

We comprehensively characterised sRNA expression in multiple myeloma patients by performing sRNA-sequencing on myeloma cells isolated from bone marrow aspirates of 86 myeloma patients. The sRNA expression profiles were correlated with the patients’ clinical data to investigate associations with survival and disease subgroups, by using cox proportional hazards (coxph) -models and *limma-voom*, respectively. A publicly available sRNA dataset was used as external validation (*n* = 151).

**Results:**

We show that multiple miRNAs are differentially expressed between ISS Stage I and III. Interestingly, we observed the downregulation of seven different U2 spliceosomal RNAs, a type of small nuclear RNAs in severe disease stages. Further, by a discovery-based approach, we identified miRNA miR-105-5p as a predictor of poor overall survival (OS) in multiple myeloma. Multivariate analysis showed that miR-105-5p predict OS independently of established disease markers.

**Conclusions:**

Overexpression of miR-105-5p in myeloma cells correlates with reduced OS, potentially improving prognostic risk stratification in multiple myeloma.

## Background

Multiple myeloma is incurable cancer arising from the clonal proliferation of terminally differentiated plasma cells in the bone marrow. Myeloma accounts for about 10% of all haematological malignancies [1]. Clinical manifestations include high levels of monoclonal antibodies in serum and urine, anaemia, multiple organ failure, immune suppression and bone disease [1]. Major improvement of treatment has increased median OS from 2.5 year in 1997 to more than 6 years as of today [2]. Today, about 16% of patients are long-term survivors with OS of more than 8 years [3], but still, nearly a quarter of patients continue to have median OS of only 2–3 years [4]. The International Staging System (ISS) has since 2005 been the standard risk stratification system for myeloma and is based on two parameters; level of serum β_2_-microglobulin which reflects tumour mass and renal function, and level of serum albumin which reflects bone marrow inflammation [5]. This score identifies three patient Groups, I, II and III, with different prognoses [1]. In 2015, the Revised-ISS (R-ISS), including also high-risk chromosomal abnormalities and serum lactate dehydrogenase levels, was introduced [6]. However, myeloma is a heterogenous disease with OS ranging from 3 months to more than 20 years. In light of the great inter-patient variability, there is need for further differentiation of patients for the use of more personalised, risk-adapted treatment approaches [7].

sRNAs are defined as non-coding RNA molecules shorter than 300 nucleotides in length [8]. miRNAs are among the most studied groups of sRNAs, and play an important role in gene regulation in human tissues [9]. miRNA biogenesis starts with transcription of a larger primary transcript in the nucleus. Following the processing by nucleases, a stem-loop-structured precursor miRNA is transported into the cytosol. The ~22 nucleotide long miRNA duplex is loaded onto a RNA-induced silencing complex (RISC) while one of the strands is degraded. The RISC complex mediates the degradation of mRNA targets through a 3´UTR complementary target site to the miRNA [9]. miRNAs repress gene expression by either mRNA cleavage or translational repression, depending on the degree of target complementarity [9]. miRNAs are frequently dysregulated in cancers due to defects in the miRNA biogenesis machinery, abnormal transcriptional regulation, epigenetic changes and amplification or deletion of miRNA genes, and may act both as tumour suppressors and oncogenes [10]. miRNAs have become increasingly popular as biomarkers for disease, mainly due to their stability in tissue and circulation and their frequent dysregulation in disease. miRNAs are easily isolated from circulation and are also more stable in formalin-fixed, paraffin-embedded (FFPE) material and other biopsy specimens than messenger RNAs (mRNAs), and may therefore be particularly suitable as biomarkers [11].

Many miRNAs are differentially expressed in myeloma patients compared to healthy controls, and miRNAs may promote tumour progression by influencing the survival and proliferation of myeloma cells [11]. In addition, miRNA expression has been linked to distinct molecular subgroups and prognosis [11]. Several studies have identified circulating miRNAs in serum and plasma as predictors of progression-free survival (PFS) and OS in myeloma [12, 13], but the cellular sources of circulating miRNAs are largely unknown. Among endogenously expressed miRNAs from primary myeloma cells, miR-15a, miR-33b, miR-17, miR-886-5p and miR-181a have been identified as prognostic factors by qPCR- and microarray-based methods [14–16]. These survival-associated miRNAs were identified after being detected as differentially expressed in multiple myeloma or between ISS subgroups and significance was not adjusted for multiple testing across all expressed miRNAs in the cell.

While miRNAs represent a highly studied class of sRNA in myeloma, other types of sRNA have been largely overlooked. In recent years, the sRNA landscape has expanded and the expression of sRNAs is shown to be more widespread than previously anticipated. sRNA-seq can detect several types of sRNAs, such as transfer RNAs (tRNAs), small nucleolar RNAs (snoRNAs), small nuclear RNAs (snRNAs), long non-coding RNAs (lncRNAs) and piwi-interacting RNAs [17]. In cancer research, there has been an undeserved lack of attention to sRNAs, which are essential in “house-keeping” processes in the cells, including RNA splicing (snRNAs) and amino-acid peptide elongation (tRNAs) and posttranslational modifications (snoRNAs) [9, 18–20]. These essential cellular processes are often dysregulated in cancer and may reflect the influence of sRNAs on tumorigenesis. An obstacle in evaluating sRNA dysregulation and differential expression in diseased vs non-diseased tissue has been the quantification of sRNA levels, due to the lack of poly(A) tails and the presence of other molecular modifications, however, current sRNA-seq technologies have proven efficient in mapping most classes of sRNAs [8, 21]. More recently, many classes of sRNAs are shown to have prognostic value and to play functional roles in cancer [8]. For example, dysregulation of snRNAs, which are important components of the spliceosome, may affect the splicing of tumour-suppressor or oncogenic transcripts [22] and have prognostic value [8].

Here, we performed comprehensive sRNA-seq on CD138 + cells isolated from bone marrow aspirates of 86 myeloma patients. We characterised differential sRNA expression between disease stages and analysed if expression of sRNAs was associated with survival. The data are available in an interactive web application that can be used to explore the relation between sRNAs and clinical parameters, including patient survival. In the current study, we found novel associations between snRNAs, miRNAs and ISS stage. Further, by a discovery-based approach, we identified miR-105-5p expression in myeloma cells as a prognostic factor for patient OS.

## Materials and methods

### Patient cohort

The patient cohort consisted of 86 multiple myeloma patients with a median age of 67 (31–88) years, all diagnosed with myeloma C90.0. The patient’s bone marrow samples were obtained at the time of diagnosis. The patients were diagnosed in the 2012–2017-year interval in Norway, and samples were stored in Biobank1, the research biobank of the Central Norway Regional Health Authority. Patients were stratified based on the original ISS system and 34.8%, 20.9%, 32.5% of the patients were classified in Stages I, II and III, respectively, while for 11.6% of patients’ ISS stage was not determined. The cytogenetic abnormalities del(17p13) and t(4;14), were evaluated by fluorescence in situ hybridisation (FISH) analysis, while bone disease were determined either by X-ray when diagnosed before 2014 (*N* = 24), or whole-body low-dose computed tomography (CT) when diagnosed after 2014 (*N* = 62). The median PFS and OS for the patient cohort were 27 and 57 months, respectively. Further details about the patients’ clinicopathological characteristics are summarised in Table 1.

**Table 1:** Clinical and histopathological characteristics of the investigated patient cohort.

	Clinical variable	Number of patients
Gender		
	Male	52
	Female	34
Age		
	Median (min–max)	67 (31–88)
Age intervals		
	31–49	6
	50–59	17
	60–69	27
	70–79	28
	80–88	8
ISS stage		
	I	30
	II	18
	III	28
	Unknown	10
del(17p13)		
	Detected	7
	Not detected	60
	Unknown	19
t(4;14)		
	Detected	9
	Not detected	54
	Unknown	23
Albumin (g/L)		
	Median (min–max)	37 (19.6–478)
Serum M-component (g/L)		
	Median (min–max)	25.3 (0–77.5)
Blood haemoglobin (g/dL)		
	Median (min–max)	11.1 (7.1–15.5)
Blood leukocytes (pr/L)		
	Median (min–max)	5.25 (2.2–19.9)
Blood thrombocytes (pr/L)		
	Median (min–max)	205 (2.29–435)
Serum creatinine (μmol/L)		
	Median (min–max)	84 (30–871)
Serum B2M (mg/L)		
	Median (min–max)	3.9 (1.6–27.9)
Blood calcium (mmol/l)		
	Median (min–max)	2.4 (1.2–3.01)
Bone disease^a^		
	Yes	49
	No	37
Overall survival		
	Median (min–max)	52 (1.7–203)
Overall survival intervals		
	<12 months	9
	>12 < 24 months	5
	>24 < 36 months	8
	>36 < 48 months	5
	>48 months	18
	Alive	41
Progression^b^		
	Median (min–max)	27 (1.7–134)
Progression invervals^b^		
	<12 months	15
	>12 < 28 months	19
	>24 months	27
	No progression	21
	Unknown	4

^a^Defined as the presence of ≥1 osteolytic lesion as determined by X-ray (when diagnosed before 2014, *N* = 24) or CT (when diagnosed after 2014, *N* = 62).

^b^Time from diagnosis. Relapse is defined as >25% increase in serum M-component or of involved/uninvolved free light chains.

The Regional Committee for Medical and Health Research Ethics (REK2011/2029) approved the study, and all patients provided written informed consent.

### Isolation of CD138 + plasma cells and patient selection

CD138 + plasma cells were isolated from bone marrow aspirates obtained at diagnosis. The cells were isolated by RoboSep automated cell separator using Human CD138 Positive Selection Kit (StemCell Technologies, Grenoble, France) [23]. The purity of plasma cell isolates as estimated by counting plasma cells on cytospins was >95%. The cells were pelleted and stored at −80 °C at the hospital biobank (Biobank1, St. Olavs University Hospital HR, Trondheim, Norway). Before the sequencing experiment, 86 samples were randomly selected from the biobank without prior knowledge of the groups and investigations performed in the current study. The sample size was chosen based on available samples in the biobank, experience from similar studies by the authors, and sample sizes form similar published studies in multiple myeloma.

### RNA isolation, library preparation and sequencing

RNA for sRNA-seq was isolated using miRVana total RNA isolation, following the protocol, (ThermoFisher, #AM1560). sRNA-seq libraries were randomly prepared from 400 ng of RNA using the NEXTFLEX Small RNA-Seq Kit v3 (PerkinElmer, #NOVA-5132-05) using 16 PCR cycles and using the thermal settings as recommended in the protocol. Ten synthetic calibrator RNAs were mixed with the input RNA during the first ligation step as previously described [24]. After library preparation, sRNAs quality and size were evaluated using Eukaryote 4 total RNA pico assay on the 2100 Bioanalyzer (Agilent Technologies) and sRNA-fragments with size larger than 140 nts (adaptors are 140 nts) and shorter than 413 nts (longest detected fragment) were excised and included in the sequencing.

Bioanalyzer result is presented in Supplementary Fig. 1C. The sequencing libraries were sequenced on the NextSeq 500 System from Illumina.

mRNA-seq was performed on the same patient samples and these data can be accessed in the web application together with the sRNA data. The methods and workflow for the sequencing and analysis of mRNA data are published and previously described [25].

### Data processing of sRNA-seq

The raw sequencing data were processed using the following procedure: quality control of the raw sequence data was performed using fastQC [26] trimming of sequence adaptors and random nucleotides from the 3’- and 5’ end of the raw sequences was performed using cutadapt-1.2.1 [27]. The trimmed sequences were collapsed with the fastx collapser tool [28] into single unique reads along with their total read count and mapped to the human (hg38) genome using bowtie2 [29] allowing for up to ten alignments per read to account for reads from duplicated miRNA loci (bowtie2 – k10), and else default parameters. MiRNA-counts were calculated using htseq-count from the HTseq python package [30]. These reads were further filtered to identify those with perfect alignment to the genome, and the total read count for mature miRNAs were then computed by summing the total read count per sequence (isomiR) overlapping each miRNA locus. Mature miRNAs and non-coding RNAs were annotated using miRBase (Release 22, 2014) and RNA Central release 17 (http://rnacentral.org), respectively. In order to compare miRNA expression between samples, read counts were normalised using the calibrator RNA normalisation factors calculated in limma, followed by counts per million (cpm) normalisation. The calibrator RNAs were not filtered prior to normalisation and the calcNormFactors in limma were calculated using the full calibrator count matrix.

### Differential expression analysis

Differentially expressed sRNAs were detected using the *limma (v3.5)* package in R with *voom* transformation [31]. Limma-voom robustly estimates the mean-variance relationship in the data and work with log-cpm normalised counts which enables statistically robust differential expression analysis. The statistical tests in limma-voom are applied after performing mean-variance normalisation, ensuring that standard statistical test such as *t* test can be applied to counts data. The count matrices were filtered to contain RNAs that were expressed with at least 1 cpm in at least 25% of the samples. The analyses were adjusted for the patient’s age and sex. *P* value adjustment was performed using Benjamini–Hochberg. The R-script used to detect differentially expressed sRNAs can be found in the Supplemental Information.

### Survival analyses

Survival analyses are performed using a coxph model in R using the *survival* package and the functions *coxph* and *Surv*. Each sRNA-class was analysed individually, and the *P* values were corrected for multiple testing across all sRNAs within each sRNA-class using the function *qvalue* in R. The discovery survival analysis was adjusted for age, sex and ISS. The R-script for survival analyses is available in Supplemental Information.

Multivariate analysis on miR-105-5p specifically was performed using coxph by including clinical parameters that were significantly associated with OS as covariates in the model, including age, sex, ISS, haemoglobin, creatine, calcium, albumin and B2M. The R-script for the multivariate analysis can be found in Supplemental Information.

### Kaplan–Meier survival curves and calculation of cutoff value

The Cutoff algorithm was applied to determine the optimal cutoff point for high- and low expression of the miRNAs. Specifically, the normalised expression matrix was uploaded to Cutoff Finder (https://molpathoheidelberg.shinyapps.io/CutoffFinder_v1/) and the cutoff values were determined using the “Fit of mixture model” method. These cutoff values were applied to both the discovery and validation dataset. For miR-105-5p, the cutoff values were High: log2cpm > 1.031 and Low: log2cpm ≤ 1.031. The survival curves were plotted and calculated using the function *ggsurvplot* within the R package *survminer*. The script can be found in Supplemental Information.

### Analysis of validation cohort

Kryukov et al. [32] performed microarray RNA profiling by using the Affymetrix GeneChip Human Gene 1.0 ST Array in CD138 + plasma cells from 151 newly diagnosed untreated multiple myeloma patients. The survival data and processed expression data from this study were downloaded from EMBL-EBI ArrayExpress (accession number: E-MTAB-1038 and E-MTAB-4032). This microarray array also contains probes for several human miRNAs. The miRNA probes were identifies using *biomaRt* in R using the annotations available at Affymetrix: http://www.affymetrix.com/support/technical/byproduct.affx?product=hugene-1_0-st-v1. The expression data for miR-105-5p were extracted and plotted using *ggsurvplot* in R as described.

## Results

### sRNA-sequencing of CD138 + cells from multiple myeloma patients

**Fig. 1:**
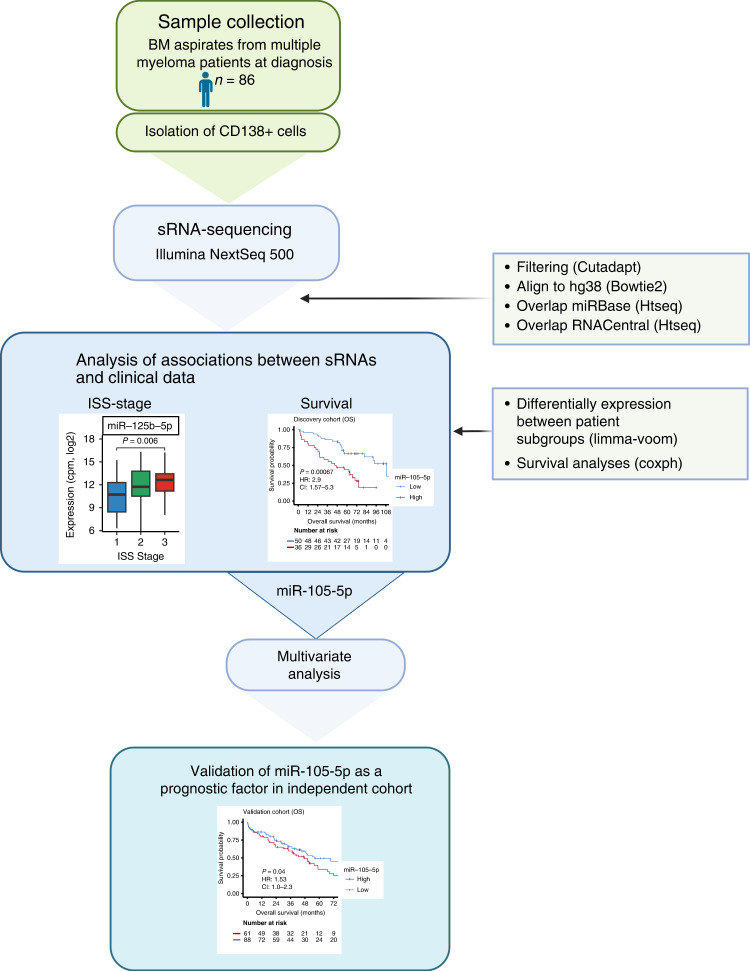
Workflow of the sRNA-seq study. Small RNAs isolated from bone marrow aspirates from 86 multiple myeloma patients were sequenced using high-throughput sequencing. MiRNAs and other small RNAs were identified and quantified by using the databases miRBase and RNACentral, respectively. The expression data was analysed with respect to the patient’s clinical data. An independent cohort with miRNA expression data was used to validate the results from the survival analysis.

**Fig. 2:**
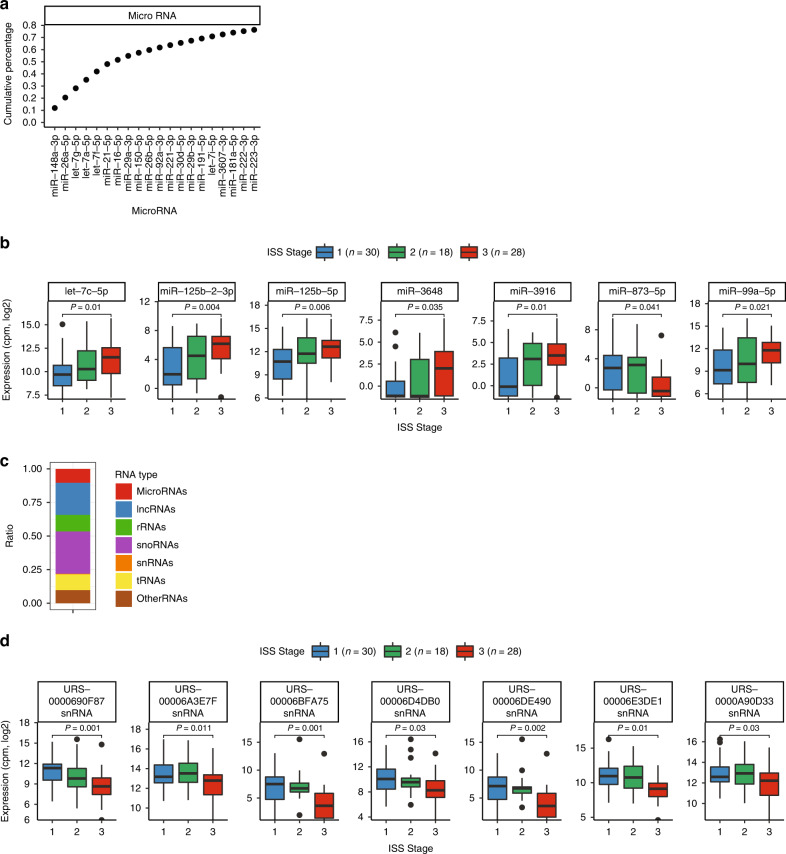
sRNA expression and its correlation with disease stage. **a** Cumulative expression of the top 20 highest expressed miRNAs. **b** Differentially expressed miRNAs between ISS Stage III and I. Bonferroni-adjusted-*P* values determined by limma in R. The number of patients within each group is shown in the legend. Note that for some patients, ISS information is lacking. **c** Relative abundance of the main sRNA classes identified, summed across all samples. **d** Differentially expressed snRNAs between ISS Stage III and I. The IDs show the RNA-type and gene-ID. See (**b**) for the explanation of the legend. The following snRNAs were located within a host gene: URS0000690F87 in gene RUVBL1; URS00006BFA75 in gene GSKIP; URS00006DE490 in gene ITF88; URS0000A90D33 in gene CCDC200.

To identify sRNA associated with disease severity and OS in multiple myeloma, we performed sRNA-seq on RNA isolated from purified bone marrow plasma cells from 86 patients at diagnosis (Table 1 and Supplementary Table S1). The main steps of the study workflow are presented in Fig. 1. On average, 54 million reads mapped to the human genome (Supplementary Fig. 1A). We detected a total of 1757 unique miRNAs and 161 of those were expressed with at least 1 count per million (cpm) in all 86 samples. The highest expressed miRNA was miR-148a-3p, and six miRNAs contributed with about 50% of the reads in the libraries (Fig. 2a).

### miRNAs differentially expressed between myeloma disease stages

We investigated if any of the expressed miRNAs were differentially expressed between patients with ISS Stage III (high-risk disease) and patients with ISS Stage I (low-risk disease). We detected seven significantly differentially expressed miRNAs, let-7c-5p, miR-125-2-3p, miR-125-5p, miR-3648, miR-3916, and miR-99a-5p, which were upregulated in Stage III compared to Stage I. One miRNA, miR-873-5p, was expressed lower in ISS Stage III compared with Stage I (Fig. 2b). For the significant miRNAs, we observed a linear trend in expression from Stage I to II to III, supporting that the expression is associated with disease severity. When adjusting for ISS stage, none of these sRNAs were associated with OS (Supplementary Table S2).

### U2 spliceosomal snRNAs are differentially expressed between myeloma disease stages

In addition to miRNAs, we detected sRNAs from several other sRNA classes. The most abundant class of sRNAs were lncRNAs, rRNAs, snoRNAs and tRNAs (Fig. 2c and Supplementary Fig. 1A, B). The distribution of sRNAs in the samples was equivalent to that observed in solid tumour cancers [33]. Motivated by the significant associations between miRNAs and ISS stage, we investigated if any of the other classes of sRNAs were differentially expressed in ISS stage. We detected significant changes in seven snRNAs, all of which showed decreased expression in Stage III compared to Stage I (Fig. 2d). All seven ISS-associated snRNAs belonged to the U2 spliceosomal RNA class, although transcribed from different genomic loci. To investigate if the changes in snRNA expression were related to changes in the transcription of the corresponding loci, we identified potential host genes of these snRNAs and investigated if the host genes were related to ISS stage. Four of the seven snRNAs were located within a host gene (see figure text in Fig. 2d), however, we did not observe any significant associations between the host gene expression and ISS stage (data for host gene expression not shown), indicating that the changes in snRNA expression are not directly linked to changes in the transcription of the corresponding loci, but rather a sum of different regulatory factors.

### miR-105-5p is associated with reduced OS in multiple myeloma

**Fig. 3:**
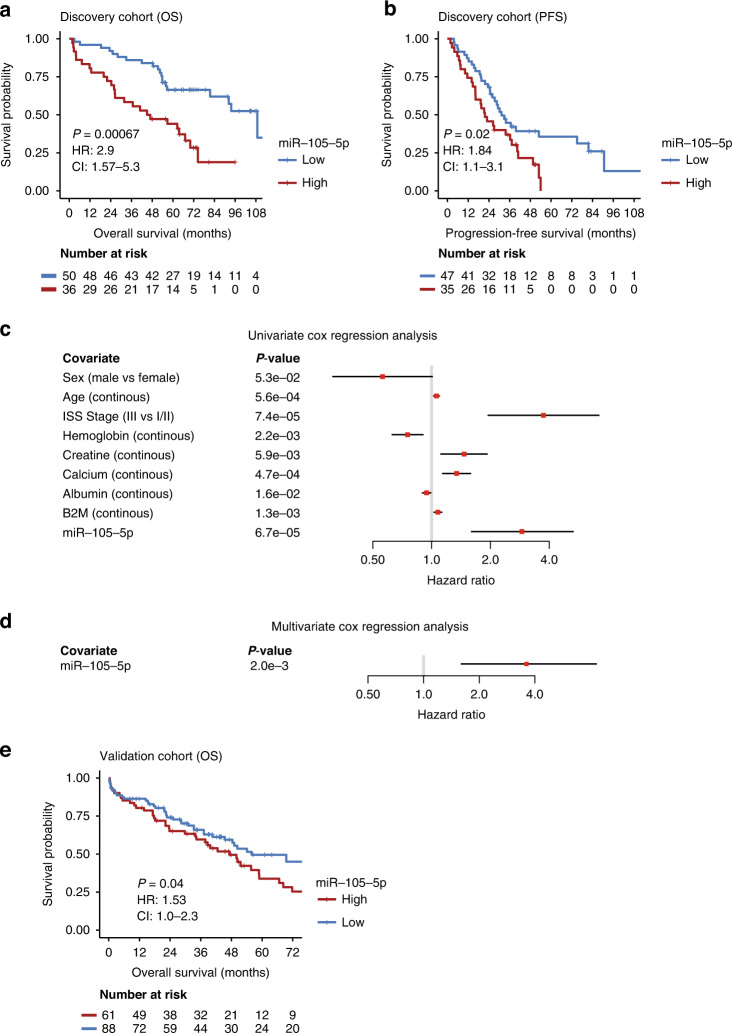
miR-105-5p associates with reduced OS. **a** Kaplan–Meier OS curves for miR-105-5p in the discovery cohort. The “+” sign on the curves indicates censored patients. The number of patients at risk at each interval is shown. At diagnosis, 36 patients had a high miR-105-5p expression, and 50 patients had low miR-105-5p expression. The *P* value is calculated by the *log-rank* test in the *coxph*-function in R. Hazard ratio and confidence interval are calculated by the *coxph*-function in R**. b** Kaplan–Meier PFS survival curves for miR-105-5p in the discovery cohort. At diagnosis, 35 patients had high miR-105-5p expression, and 47 patients had low miR-105-5p expression. Statistics calculated as described in **a**). **c** Forest plots for the univariate cox regression analyses of clinical parameters with respect to OS. Shown are parameters that were detected as significant, in addition to age which was not detected as significant. Statistics calculated as described in **a**). **d** Multivariate cox regression analysis of miR-105-5p with respect to OS adjusted for age, sex, ISS stage, haemoglobin, creatine, calcium, albumin and beta-2 microglobulin (B2M). Statistics calculated as described in **a**). **e** Kaplan–Meier OS curve for miR-105-5p in the validation cohort of the Kruykov et al. [32] Statistics calculated as described in **a**).

Having shown that multiple miRNAs and other sRNAs are associated with the myeloma disease stage, we investigated if miRNAs or other sRNAs can predict OS and PFS. Using cox proportional hazards regression (coxph) with adjustment for multiple testing across all expressed miRNAs and likewise for the other sRNAs, we detected one miRNA, miR-105-5p, to be significantly associated with OS. No other miRNA or sRNA were significantly associated with PFS or OS after adjustment for multiple testing. High expression of miR-105-5p was associated with shorter OS (HR: 2.9; 95% CI: 1.57–5.3; *P* value: 0.00067 and 0.02 before and after correcting for multiple testing, respectively). The optimal cutoff point for high and low miR-105-5p was calculated using Cutoff Finder [34], which split the data at the 57th percentile, resulting in 36 patients in the high-group (log2cpm > 1.031) and 50 patients (log2cpm ≤1.031) in the low-group (Supplementary Fig. 2A). The Kaplan–Meier survival curves for miR-105-5p showed clear differences in median survival between the groups (3313 days for the “miR-105-5p low” group and 1398 days for the “miR-105-5p high” group) (Fig. 3a). MiR-105-5p was also associated with PFS (HR: 1.84; 95% CI: 1.1–3.1; *P* value: 0.02) (Fig. 3b). Next, we performed OS univariate coxph for the available clinical parameters for the patient cohort. We identified seven clinical parameters to be significantly associated with OS of which age, ISS stage, creatine, calcium and beta-2 microglobulin (B2M) were negatively associated with OS (HR > 1) and Haemoglobin and Albumin were positively associated with OS (HR < 1) (Fig. 3c). We did not find any significant association with the two available high-risk cytogenetic aberrations, deletion 17p13 and translocation (4;14), most likely due to small N (Supplementary Fig. 2B, C). We then evaluated the independent prognostic value of miR-105-5p with respect to OS by adjusting for age, sex, ISS stage, haemoglobin, creatine, calcium, albumin and B2M in a coxph model. The multivariate OS model showed that miR-105-5p retained its prognostic value (HR: 3.6; 95% CI: 1.56–8.5; *P* value: 0.002) (Fig. 3d). Combining miR-105-5p expression and ISS groups in a coxph-analysis showed that patients with high miR-105-5p and high ISS (ISS III) had significantly poorer survival compared to patients with low miR-105-5p and high ISS (HR: 4.98; 95% CI: 1.65–15; *P* value: 0.004) (Supplementary Fig. 3A and Supplementary Table S3). The patients with ISS III and high miR-105-5p had median OS of 804 days, while the patients with ISS III and low miR-105-5p lived 2480 days, indicating that miR-105-5p distinguish the high-risk patients. The multivariate PFS survival model was, however, not significant (HR: 1.85; CI: 0.9–3.8; *P* value: 0.09), although pointed in the same direction as the univariate PFS survival model.

To validate the prognostic value of miR-105-5p, we analysed the microarray dataset of Kryukov et al. (*n* = 149) [32]. Supporting the results from the discovery dataset, high expression of miR-105-5p was associated with unfavourable OS in the validation cohort (HR: 1.53; 95% CI: 1–2.3; *P* value: 0.04) (Fig. 3e). PFS survival of miR-105-5p was not significant in the validation cohort (*P* value: 0.6).

## Discussion

Myeloma is a heterogenous cancer with large differences in survival between patients. Parameters for risk stratification include (R)-ISS staging, cytogenetics and other host factors (such as age). ISS staging combined with high-risk cytogenetics features /R-ISS is the standard system in routinely clinical use [1]. The implementation of chromosomal abnormalities in the ISS risk evaluation system has substantially improved the prediction of patient survival, but even within R-ISS subgroups there are further risk heterogeneity [7]. Identifying all high-risk myeloma patients is still a challenge and there is still room for improvement of the current stratification system. Precise risk prediction at diagnosis enables early intervention and opens for the use of risk-adapted treatment approaches and inclusion in clinical trials [35, 7].

In this study, we compared the expression of sRNAs in primary myeloma cells obtained from different disease stages and evaluated the association of sRNAs with patient prognosis. We found one miRNA, miR-105-5p to be an independent prognostic marker for poor OS in myeloma. sRNAs are promising biomarkers, predominantly studied in liquid biopsies [36]. With miRNAs as an exception [37], there is limited knowledge about this important group of non-coding RNAs in myeloma. The limited number of sRNA-seq studies may explain this knowledge gap. Previously, a sRNA-seq study focusing on miRNA and miRNA-offset-RNA was conducted on 30 myeloma patients [38]. MiRNA-offset-RNAs, which are stable, sRNA molecules with unknown functions transcribed from different miRNA loci [39], were found to associate with patient molecular subgroups in myeloma [38]. However, the characterisation of other sRNA species in the samples and how sRNA expression associated with survival were not investigated [38].

Here, using sRNA-seq, we identified snRNAs and miRNAs differentially expressed in disease stages of myeloma. With an exception for miR-873-5p, which was downregulated in Stage III compared to Stage I, expression of all the significant miRNAs, including let-7c-5p, miR-125-2-3p, miR-125b-5p, miR-3648, miR-3916, and miR-99a-5p was increased with increased severity of the disease. The U2 snRNAs, on the other hand, were downregulated in ISS III compared with ISS I, suggesting that reduction in mRNA splicing may be an oncogenic driver. None of the miRNAs and only some of the snRNAs that were associated with ISS stage are located within an annotated host gene. Moreover, those that were intragenic were not associated with ISS stage. This indicates that the differential expression of sRNA with ISS stage is not a direct effect of changes in host gene expression but may instead be directly related to their function in disease.

snRNAs have recently been identified as an important class of sRNAs that contributes to genome stability and in maintaining correct mRNA splicing [40]. The U2 snRNAs is a crucial component in the U2 small nuclear ribonucleoprotein, which is part of the spliceosome complex that remove introns from eukaryotic precursor mRNAs. The U2 snRNAs, which are important for both spliceosome assembly, intron substrate recognition and protein scaffolding are thought to play a role in cancer [41]. Knockdown experiments with snRNAs in breast cancer led to alternative splicing of genes frequently dysregulated in breast cancer, pointing towards a role of snRNAs as global gene regulators [22]. U2 knockdown primarily affected mRNA splice site recognition and exon inclusion [22]. The impact of U2 spliceosomal snRNAs expression has not previously been characterised in myeloma, but aberrant RNA splicing has recently been related to high-risk molecular subgroups and poor prognosis [42].

Previous miRNA studies that have investigated survival in myeloma have selected their miRNAs based on disease-related expression patterns such as differential expression in myeloma or between ISS stages, and subsequently evaluated their prognostic value. In our study, we identified all expressed miRNAs by sRNA-seq and evaluated their prognostic significance by discovery-based survival analysis, without a priori selection of specific miRNAs. A limitation of this study is the number of patients sequenced. Increasing the number of subjects would most likely increase the number of significant discoveries as many sRNAs were significantly associated with survival before adjusting for multiple testing.

Considering this, miR-105-5p stands out as a robust prognostic factor that predicts reduced survival, despite low *N*. Also, miR-105-5p significantly predicted survival in a multivariate model, indicating that it has an independent prognostic value. Finally, miR-105-5p is a prognostic marker in another, independent dataset, supporting the validity of our finding.

In our patient cohort the median OS time for patients with high miR-105-5p was 3.8 years, while for the patients with low miR-105-5p median survival was 9.07 year, thus miR-105-5p can be used to distinguish high-risk patients from low-risk patients. Furthermore, within the ISS III patient group, high miR-105-5p expressors had significantly worse outcome compared to low miR-105-5p expressors, suggesting that miR-105-5p may be used for further discrimination of patients in the high-risk subgroup. Patients with ISS III and high miR-105-5p had a median OS of 2.2 years while patients with ISS III and low miR-105-5p that had a median OS of 6.8 years, thus miR-105-5p may be a marker that can further stratify ultra-high-risk [43] patients from the other high-risk patients. Our dataset includes patients diagnosed in the years 2012–2017, and t(14;16) and LDH measurements were not available for the majority of patients. Thus, the patient’s disease stage is therefore based on the “original” and not the R-ISS system, which limits the possibility of investigating miR-105-5p with respect to the R-ISS.

In summary, we provide a comprehensive characterisation of sRNAs in primary cells from myeloma patients and identify U2 spliceosomal snRNAs and miR-105-5p as novel potential biomarkers for disease stage and patient survival, respectively. The prognostic value of miR-105-5p was validated in an independent dataset, and we suggest further evaluation of miR-105-5p as a stratification parameter in future studies on myeloma patients to determine the clinical relevance as a biomarker for high-risk disease. Our study also warrants further investigation of the functional roles of U2 spliceosomal snRNAs and miR-105-5p in the pathogenesis of multiple myeloma.

## Supplementary information

Supplementary information

Supplementary Figure 1

Supplementary Figure 2

Supplementary Figure 3

Supplementary Table 1

Supplementary Table 2

Supplementary Table 3

aj-checklist

## Data Availability

The sRNA data and a comprehensive set of clinical data are available through an interactive web application, on which additional associations can be investigated (https://github.com/MjelleLab/MicroRNA-and-Gene-Expression-In-Multiple-Myeloma). The processed count matrices for all RNA classes, as well as the clinical data, are available on the same site. mRNA-miRNA, mRNA-clinical associations can also be assessed, as we have mRNA-seq data from the same samples [25]. Due to Norwegian law on sensitive data, raw data cannot be submitted to public repositories, however, all raw data are available upon request to the corresponding author.

## Code availability

The R code for statistical analysis is found in the supplemental information.
