# Supplementary material for: Comprehensive small RNA-sequencing of primary myeloma cells identifies miR-105-5p as a predictor of patient survival
Source: Br J Cancer. 2022 Nov 29;128(4):656–64. doi: 10.1038/s41416-022-02065-1 (PMC9938247; doi:10.1038/s41416-022-02065-1)
Supplement: Supplementary file 1 — Supplementary information [file 41416_2022_2065_MOESM1_ESM.docx]

**Comprehensive small RNA-sequencing of primary myeloma cells identifies miR-105-5p as a predictor of patient survival**

Kristin Roseth Aass^1,2^, Tonje Marie Vikene Nedal^1,2^, Siri Anshushaug Bouma^2^, Synne Stokke Tryggestad^1,2^, Einar Haukås^3^, Tobias Schmidt Slørdahl^2,4^, Anders Waage ^2,4^, Therese Standal^1,2,4#^, Robin Mjelle^2,5,6#^

# Shared senior authorship

^1^Centre of Molecular Inflammation Research, Department of Clinical and Molecular Medicine, Norwegian University of Science and Technology, 7491, Trondheim, Norway

^2^ Department of Clinical and Molecular Medicine, Norwegian University of Science and Technology,7491, Trondheim, Norway

^3^ Department of Hematology, Stavanger University Hospital, 4011, Stavanger, Norway

^4^ Department of Hematology, St. Olavs University Hospital, 7030, Trondheim, Norway

^5^ Bioinformatics Core Facility - BioCore, Norwegian University of Science and Technology NTNU, 7491 Trondheim, Norway

^6^ Department of Pathology, St. Olavs University Hospital, 7030, Trondheim, Norway

**Supplemental method descriptions**

**Differential expression analysis**

The following R-script was used to detect differentially expressed sRNAs:

*expr.mat.dge <- edgeR::DGEList(counts = expr.mat)*

*keep <- rowSums(edgeR::cpm(expr.mat.dge)>1) >= dim(expr.mat.dge)[2]/4*

*expr.mat.dge <- expr.mat.dge[keep,]*

*expr.mat.calibrator.dge <- DGEList(expr.mat.calibrator)*

*expr.mat.calibrator.dge <- calcNormFactors(expr.mat.calibrator.dge)*

*expr.mat.calibrator.dge <- calcNormFactors(expr.mat.calibrator.dge, method="TMM")*

*expr.mat.dge$samples$norm.factors <- expr.mat.calibrator.dge$samples$norm.factors*

*des <- model.matrix(~0+factor(ISS),+Age+Sex, exclude=NULL))*

*colnames(des) <- c("ISS1","ISS2","ISS3","Unknown")*

*v <- voom(Mature_Standal_Biobank.dge,design = des)*

*fit <- lmFit(v, design=des)*

*contrasts <- makeContrasts(cond1=ISS3-ISS1, levels=des)*

*fit2 <- contrasts.fit(fit, contrasts=contrasts)*

*fit2 <- eBayes(fit2)*

*colSums(decideTests(fit2)!=0)*

*colSums(decideTests(fit2, method="glob")!=0)*

*topTable1 <- topTable(fit2,coef="cond1",sort.by="P",adjust.method="BH", n=10)*

*expr.mat is the miRNA/sRNA expression matrix*

*expr.mat.calibrator is the calibrator expression matrix*

**Survival analyses**

Survival analyses are performed using a coxph-model in R using the *survival* package and the functions *coxph* and *Surv* . The following R-script was used for discovery assessment of each sRNA class:

*coxph.sRNA_ <- capture.output(for(i in colnames(data.df)){print(summary(coxph(as.formula(paste0("Surv(Time, Status)~", i, "+Age","+Sex",+”ISS”)), data=as.data.frame(data.df)))) })*

*data.df* is the input data frame containing expression values and clinical parameters:

*Time* is the Survival time

*Status* is the status of the patients (dead/alive)

*Age* is the age of the patient

*Sex* is the sex of the patient

*ISS* is the ISS stage

*i* is the tested sRNA

The following *coxph*-model was used for the multivariate analysis:

coxph(Surv(Time, Status) ~miR_105_5p+Age+Sex+ISS+Hemoglobin+ Creatine+ Calsium+ Albumin+ Beta2_Microglobulin, data = as.data.frame((data.df)))

**Kaplan–Meier survival curves**

The survival curves and p-values were plotted and calculated using the function *ggsurvplot* within the R package *survminer* using the following script:

*p <- ggsurvplot(fitSurv.miRNA ,title="", xlab="Time (Yrs)", ylab="Overall survival probability", font.main = 8, font.x = 8,font.y = 8,font.tickslab = 8,font.legend=8,pval.size = 2,pval.coord = c(50,0.3), size=0.4,legend = "right",censor.size=2,break.time.by = 365, pval ="", palette = c("#3182bd", "#de2d26"), ggtheme = theme_classic(),risk.table = T, fontsize = 2 , tables.theme = theme_cleantable(), xscale=365.25, xlim=c(0,7*365), legend.title = "",legend.labs = c(""))*

*p$table <- p$table + theme(plot.title = element_text(size = 8, color = "black", face = "bold"),axis.text.y = element_blank(),axis.ticks.y = element_line(colour = c("#de2d26","#3182bd"),size=1),axis.ticks.length=unit(.4, "cm"))*

*fitSurv.miRNA* is the survfit object

**References:**

1. Mjelle, R., et al., *Identification of metastasis-associated microRNAs in serum from rectal cancer patients.* Oncotarget, 2017. **8**(52): p. 90077-90089.

2. Kryukov, F., et al., *Centrosome associated genes pattern for risk sub-stratification in multiple myeloma.* J Transl Med, 2016. **14**(1): p. 150.

**Supplementary Figure S1: Small RNA sequencing statistics. A)** The panel “Library size” shows the total library size. The panel “Alignments” shows the alignment-statistics, including reads that align at one position in the genome (SingleAligned); reads that align at multiple positions in the genome (MultiAligned); and reads that do not align to the human genome (NotAligned). The panel “Features” shows reads that overlap either the miRNA database miRBase, or the database of non-coding RNAs, RNACentral. The panel “RNAs” shows the absolute expression of the main RNA-classes detected. **B)** Shown is the relative expression of the main RNA-classes detected. **C)** A reprehensive bioanalyzer result for the miRNA libraries. Shown is the length of the adapter-ligated RNA fragments after small RNA sequencing library preparation. The “miRNA-peak” has a length of 151 nucleotides (due to adapters). The peaks longer than the miRNA-peak represent snoRNA, tRNAs and other non-coding RNAs.

**Supplementary Figure S2:** Results from Cutoff Finder and survival curves. **A)** Histogram of miR-105-5p expression values. A mixture model of two Gaussian distributions is fitted to each of the histograms (red lines). Vertical lines designate the optimal cutoffs derived from the mixture model using Cutoff Finder. **B)** Survival curves for patients with t(4;14) translocation (n=9) and patients without t(4;15) translocation (n=54). **C)** Survival curves for patients with 17p13 deletion (n=7) and patients without 17p13 deletion (n=60).

**Supplementary Figure S3:** Survival curves for miR-105-5p. **A)** Survival curves for patients with high and low miR-105-5p in combination with ISS stage.
