# Supplementary figures and images for: Comprehensive small RNA-sequencing of primary myeloma cells identifies miR-105-5p as a predictor of patient survival
Source: Br J Cancer. 2022 Nov 29;128(4):656–64. doi: 10.1038/s41416-022-02065-1 (PMC9938247; doi:10.1038/s41416-022-02065-1)

# Supplementary Figure 1

A

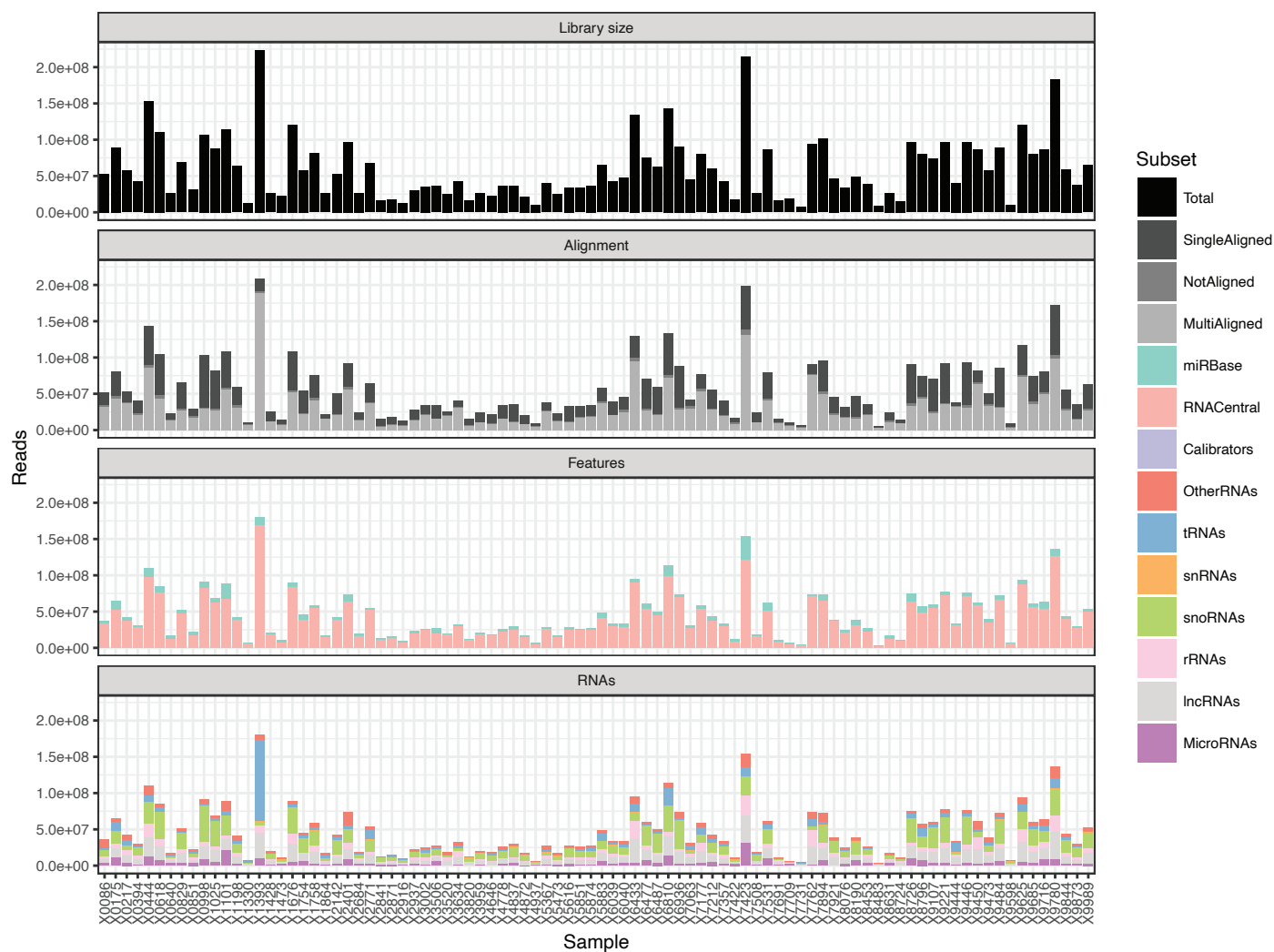

B

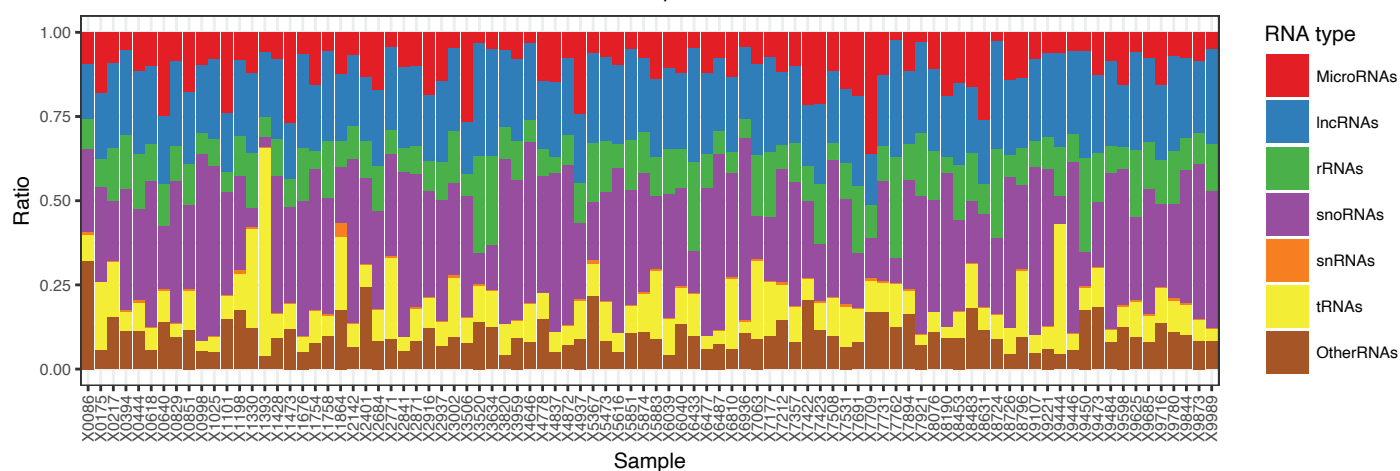

C

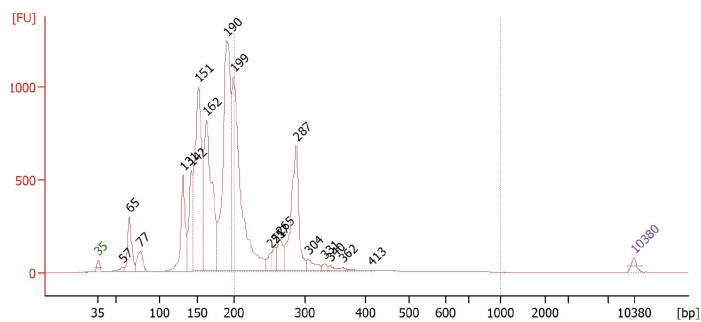

Supplement: Supplementary file 2 — Supplementary Figure 1 [file 41416_2022_2065_MOESM2_ESM.pdf]

Supplementary Figure 2

A

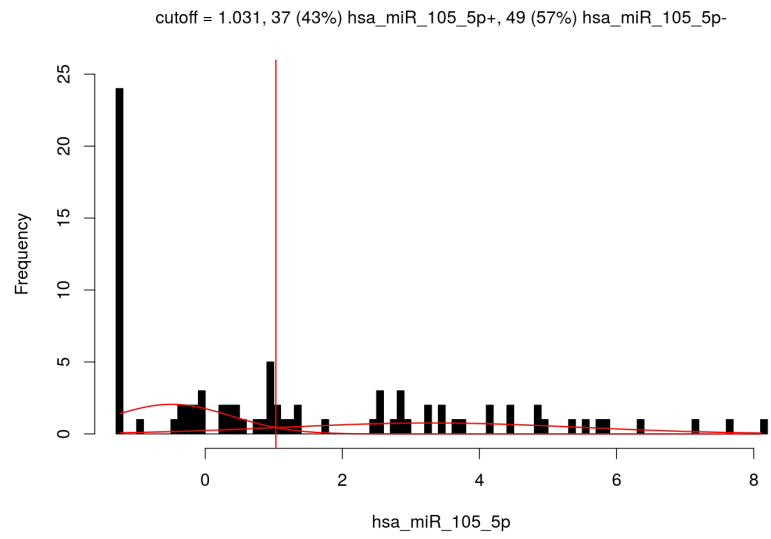

B

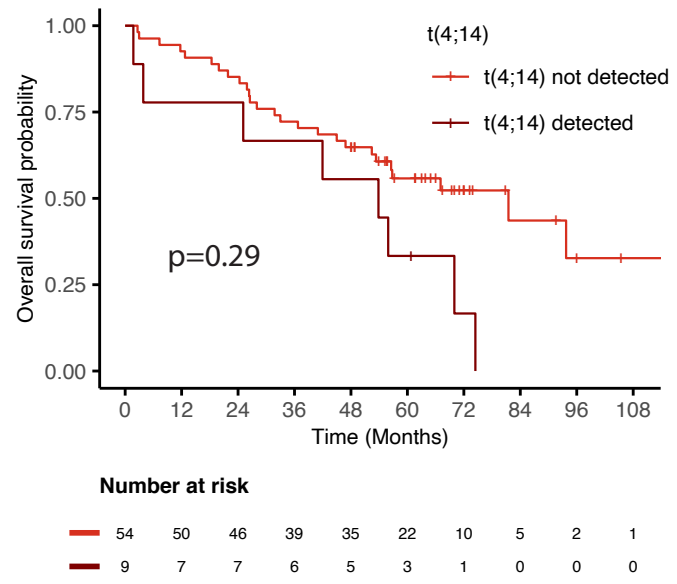

C

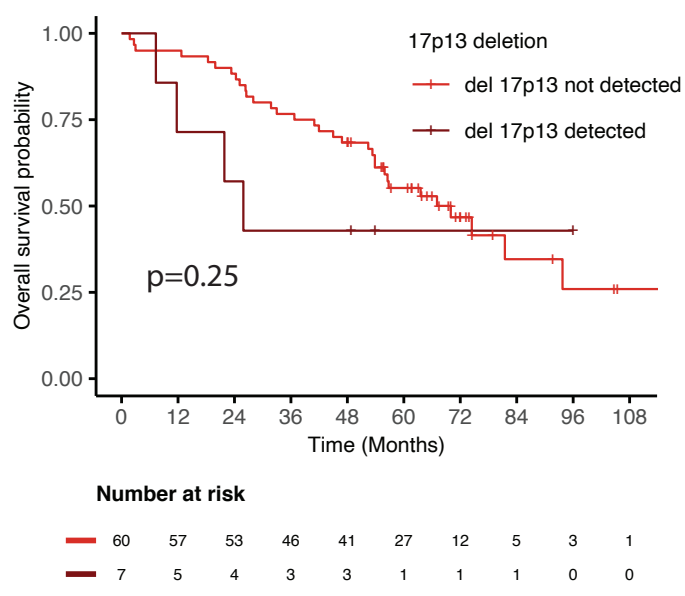

Supplement: Supplementary file 3 — Supplementary Figure 2 [file 41416_2022_2065_MOESM3_ESM.pdf]

# Supplementary Figure 3

A

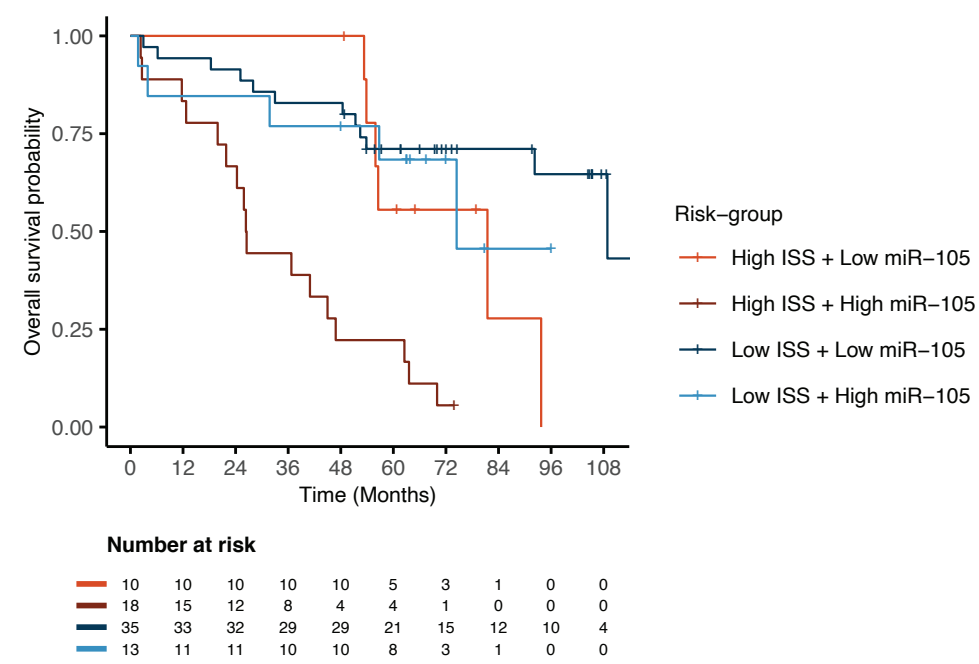

Supplement: Supplementary file 4 — Supplementary Figure 3 [file 41416_2022_2065_MOESM4_ESM.pdf]
